# Single-Cell Transcriptomic Identification of a Proliferative Intermediate State During Early Anagen Hair Follicle Regeneration

**DOI:** 10.3390/genes17091058

**Published:** 2026-08-31

**Authors:** Yueyan Liu, Xusheng Wang, Juan Wen

**Affiliations:** 1 School of Pharmaceutical Sciences, Sun Yat-sen University, Shenzhen 518107, China; liuyy588@mail2.sysu.edu.cn (Y.L.); wangxsh27@mail.sysu.edu.cn (X.W.); 2 The Seventh Affiliated Hospital, Sun Yat-sen University, Shenzhen 518107, China

**Keywords:** hair follicle regeneration, secondary hair germ, single-cell RNA sequencing, proliferative intermediate state, cell-state transition, anagen

## Abstract

**Background:** Secondary hair germ (SHG)-associated epithelial cells are a key progenitor population that contribute to hair follicle regeneration during early anagen. Although SHG-associated cells become activated during early anagen and contribute to the formation of regenerating follicular structures, the morphologically recognizable SHG compartment becomes less apparent as lower hair follicle structures develop during mid-anagen. However, the transcriptional changes accompanying the transition from SHG-associated states toward lower hair follicle-associated states remain poorly characterized. This study aimed to characterize the transcriptional changes associated with this transition and identify a proliferative intermediate transcriptional state during early anagen. By integrating single-cell transcriptomic analysis with regulatory and cell–cell communication analyses, we sought to improve the understanding of cellular state transitions and microenvironmental changes during early hair follicle regeneration. **Methods:** Hematoxylin and eosin staining (H&E) was performed to examine histological changes in mouse dorsal skin on days 0, 5, 10, 15, 20, 25, and 30 of the hair cycle. In parallel, previously published single-cell RNA sequencing data from mouse dorsal skin during early anagen were reanalyzed. Hair follicle epithelial cells were extracted and reclustered to identify cell subpopulations with distinct transcriptional characteristics. Cell types were annotated based on canonical marker genes and subpopulation-specific expression patterns. Differential expression analysis, functional enrichment analysis, cell-cycle analysis, developmental potential assessment, trajectory inference, transcription factor regulon analysis, and cell–cell communication analysis were further performed to characterize the relationships among hair germ cells, proliferative lower hair follicle-like cells (PR-loHF), and lower hair follicle cells. **Results**: Proliferative lower hair follicle-like cells (PR-loHF) were identified as a transcriptionally defined intermediate state associated with the transition from secondary hair germ (SHG)-associated cells toward lower hair follicle-associated cells. PR-loHF exhibited enrichment of biological programs associated with cell-cycle activity, epithelial remodeling, extracellular matrix organization, and developmental regulation. Moreover, compared with SHG-associated and lower hair follicle-associated states, PR-loHF exhibited distinct transcription factor regulon activity and cell–cell communication patterns, further supporting its characterization as a transcriptionally distinguishable intermediate state. **Conclusions:** PR-loHF represents a proliferative intermediate transcriptional state emerging during the transition from secondary hair germ (SHG)-associated cells toward lower hair follicle-associated states, providing new insights into transcriptional transitions and microenvironmental regulation during early hair follicle regeneration.

## 1. Introduction

The hair follicle is a highly dynamic mini-organ that undergoes repeated cycles of growth (anagen), regression (catagen), and relative quiescence (telogen) throughout adult life [1,2]. During catagen, most of the lower portion of the hair follicle undergoes degeneration, whereas epithelial stem and progenitor cells located within the permanent follicular compartment are retained. At the onset of a new anagen phase, these cells are reactivated and generate rapidly proliferating progeny that reconstruct the lower hair follicle and subsequently produce the inner root sheath and hair shaft [1,2,3,4]. This regenerative process requires the coordinated regulation of cell proliferation, lineage specification, epithelial remodeling, and interactions between epithelial cells and their surrounding microenvironment.

Secondary hair germ (SHG)-associated epithelial cells constitute a small progenitor population located below the bulge and adjacent to the dermal papilla during telogen. Rather than remaining completely quiescent, SHG-associated cells respond rapidly to activating signals at the telogen-to-anagen transition and proliferate before the major activation of bulge hair follicle stem cells [5,6]. This sequential activation has led to a two-step model of hair follicle regeneration in which SHG-associated cells contribute to early follicular growth, followed by the contribution of bulge stem cells and their descendants to the expanding follicle [5]. Intravital imaging and lineage-tracing studies have further demonstrated that the spatial position of epithelial progenitors relative to the dermal papilla influences their activation and contribution to the regenerating lower follicle [6,7]. In addition, Lgr5-positive cells distributed within the secondary hair germ (SHG) and lower outer root sheath exhibit proliferative activity, long-term persistence, and multilineage potential, highlighting the developmental relationship between early progenitor populations and the lower hair follicle [8].

The activation and transcriptional remodeling of SHG-associated cells and hair follicle stem cells are controlled by reciprocal interactions with the local niche. Dermal papilla-derived activating signals must overcome inhibitory factors within the surrounding microenvironment before anagen can begin. In particular, coordinated changes in Wnt/β-catenin, bone morphogenetic protein, transforming growth factor-β, and Sonic hedgehog signaling regulate stem-cell activation, progenitor expansion, and lower follicle formation [5,6,9,10]. As anagen progresses, epithelial progenitors also undergo changes in cell adhesion, extracellular matrix interactions, and communication with mesenchymal and other skin cell populations [10,11]. These findings indicate that early hair follicle regeneration is not driven by a single cell type or signaling pathway, but instead depends on coordinated changes in epithelial cell states and their microenvironment.

Despite substantial progress in defining the initiation of anagen, the transcriptional changes accompanying the transition of SHG-associated cells toward lower hair follicle formation remain incompletely resolved. During the transition from early to mid-anagen, the morphologically distinguishable SHG compartment gradually loses its defined boundary as the follicular epithelium extends downward and the lower hair follicle becomes established. Although previous lineage-tracing and imaging studies support the contribution of early epithelial progenitors to follicular regeneration [5,6,7,8], they do not fully resolve the transcriptional states through which SHG-associated cells acquire lower hair follicle characteristics. In particular, the transcriptional intermediate states that emerge during the transition from SHG-associated cells toward lower hair follicle-associated states, together with their molecular programs and microenvironmental interactions, remain insufficiently characterized.

Single-cell RNA sequencing has provided an effective approach for resolving cellular heterogeneity and dynamic cell states within the skin and hair follicle. Previous single-cell studies have identified transcriptionally distinct epithelial populations along the spatial axis of the hair follicle and demonstrated that hair follicle identity is shaped by both differentiation status and anatomical location [12]. Hair-cycle-dependent analyses have further revealed extensive remodeling of epithelial, mesenchymal, and immune cell states during hair growth and rest [13]. Moreover, single-cell analyses of developing follicles have identified signaling networks among stem-cell precursors, proliferative progenitors, differentiating epithelial cells, and their mesenchymal niches [11,14]. Nevertheless, the transcriptional relationship between SHG-associated states and lower hair follicle states during early anagen has not been fully characterized, and the intermediate states potentially involved in this transition remain poorly defined.

In the present study, we combined histological observations across the mouse hair cycle with a focused reanalysis of single-cell RNA sequencing data from mouse dorsal skin during early anagen. We extracted and reclustered hair follicle epithelial cells to investigate the transcriptional relationship between SHG-associated states and lower hair follicle cell states. We identified a proliferative lower hair follicle-like transcriptional state, termed PR-loHF, which exhibited strong cell-cycle activity together with lower hair follicle, epithelial adhesion, extracellular matrix, and developmental transcriptional features. Graph-based connectivity, developmental potential assessment, and trajectory inference positioned PR-loHF between SHG-associated and lower hair follicle-associated transcriptional states along an inferred continuum. We further characterized its biological functions, transcription factor regulon activity, and cell–cell communication patterns. These results support PR-loHF as a transcriptionally defined proliferative intermediate state associated with the transition between SHG-associated and lower hair follicle-associated states.

## 2. Materials and Methods

### 2.1. Histological Processing and Hematoxylin and Eosin Staining

Eight-week-old female C57BL/6 mice were used in this study. To synchronize the hair cycle, dorsal hair was removed using an electric clipper followed by depilatory cream treatment. Mouse dorsal skin samples were collected on days 0, 5, 10, 15, 20, 25, and 30 of the hair cycle. After removal of the dorsal hair using an electric clipper and depilatory cream, skin tissues were excised parallel to the vertebral line, flattened, and fixed in pre-cooled 4% paraformaldehyde at 4 °C for 24 h. The samples were subsequently dehydrated through a graded ethanol series, cleared, infiltrated with paraffin, and embedded with the skin surface oriented perpendicular to the bottom of the embedding mold to obtain longitudinal sections along the hair follicle axis. Paraffin-embedded tissues were sectioned at a thickness of 4 μm.

The sections were deparaffinized, rehydrated through a graded ethanol series, and stained with hematoxylin and eosin according to standard procedures. After staining, the sections were dehydrated, cleared, and mounted using a neutral resin mounting medium. The stained sections were digitally scanned at 20× magnification using a Jiangfeng digital pathology slide scanner (Jiangfeng Bio, Ningbo, China). Histological changes at different time points were evaluated according to overall hair follicle morphology, follicle length, dermal papilla position, and the structural characteristics of the hair germ and lower hair follicle.

### 2.2. Single-Cell RNA Sequencing Data Acquisition

The single-cell RNA sequencing data analyzed in this study were generated in a previously published study entitled *“Intensive Stress Impedes Hair Follicle Growth through Triggering Cell Cycle Arrest of Hair Follicle Stem Cells”* [15]. In the original study, three 8-week-old female C57BL/6 mice were used in the control group analyzed in the present study. Hair follicle regeneration was synchronized by depilation, and dorsal skin tissues were collected 7 days after depilation, corresponding to early anagen. The present study included only samples from the control group, whereas samples from the stress-treated group were excluded.

In the original study, single-cell suspensions prepared from mouse dorsal skin were used for library construction with the Chromium Single Cell 3′ Gene Expression v3 platform (10x Genomics, Pleasanton, CA, USA). The libraries were subsequently sequenced on an Illumina NovaSeq 6000 platform (Illumina, San Diego, CA, USA) using a paired-end 150 bp sequencing strategy. The processed gene expression matrix and corresponding cell-level metadata were obtained directly from the authors of the original study and used for the subsequent bioinformatic analyses. The data analyzed in the present study have not been deposited in a public repository. Further details regarding animal treatment, depilation-induced hair regeneration, tissue dissociation, single-cell library construction, sequencing, and initial data processing are provided in the original publication [15].

### 2.3. Single-Cell RNA Sequencing Data Processing and Cell Clustering

The processed gene expression matrix and corresponding cell metadata were analyzed in R (version [4.3.0]) using the Seurat package (version [5.0.3]) [16]. Doublets were identified and removed using DoubletFinder (version [2.0.5]) [17]. Cells with fewer than 200 or more than 8000 detected genes, more than 50,000 unique molecular identifiers (UMIs), or a mitochondrial transcript proportion greater than 25% were excluded. After application of these quality-control criteria, a total of 6618 cells with qualified transcriptomic profiles were retained for downstream analysis. Gene expression data were normalized using the LogNormalize method with a scale factor of 10,000. Highly variable genes were identified, followed by data scaling and principal component analysis.

A shared nearest-neighbor graph was constructed based on the selected principal components, and cells were clustered using the Louvain algorithm [18] as implemented in Seurat. Uniform manifold approximation and projection (UMAP) was used to visualize the transcriptional relationships among cells [19]. Major skin cell populations were annotated according to canonical marker genes and cluster-specific expression patterns [12,13].

Following annotation of the major skin cell populations, 4196 epithelial cells, representing 63.4% of the 6618 quality-controlled cells, were identified and extracted for further analysis. Among these, 383 cells were annotated as keratinocytes (KC), whereas the remaining 3813 cells were assigned to hair follicle-associated epithelial populations, corresponding to 57.6% of all quality-controlled cells. These cells were independently reanalyzed and subjected to normalization, highly variable gene selection, data scaling, principal component analysis, shared nearest-neighbor graph construction, and UMAP visualization. Graph-based reclustering was performed at a resolution of 0.6. Based on canonical marker genes, cluster-enriched genes, and overall transcriptional characteristics, the epithelial populations were annotated as keratinocytes (KC), upper hair follicle cells (uHF), lower hair follicle cells (loHF), proliferative lower hair follicle-like cells (PR-loHF), secondary hair germ cells (SHG), inner bulge cells (IB), inner root sheath cells (IRS), and medulla/cortex/cuticle cells (ME_CO_CU) [12,13].

### 2.4. Trajectory Inference and Developmental Potential Analysis

Trajectory analyses were performed on SHG, PR-loHF, and loHF cells to investigate their potential transcriptional relationships. Based on the established role of secondary hair germ (SHG) cells during anagen initiation, SHG were used as the starting population for trajectory analyses when a predefined root was required.

Partition-based graph abstraction (PAGA) was performed using Scanpy (version 1.6.0) to evaluate the topological connectivity among SHG, PR-loHF, and loHF populations [20]. Cluster-level connectivity was calculated to construct the PAGA graph, and diffusion pseudotime was estimated after manually specifying SHG cells as the starting population.

Slingshot (version [2.2.0]) was applied to infer lineage structure and pseudotime based on the reduced-dimensional representation and predefined cell clusters [21]. A minimum spanning tree was constructed among the cell populations, followed by the fitting of simultaneous principal curves to estimate lineage trajectories and cell-level pseudotime values.

Monocle 3 (version [1.3.1]) was additionally used to reconstruct the principal graph in UMAP space and independently evaluate the inferred cell-state transition [22]. After preprocessing, dimensionality reduction, and graph learning, SHG were selected as the trajectory root, and pseudotime values were calculated and projected onto the UMAP representation.

Cellular dynamics were further evaluated using scTour (version [1.0.0]), a deep-learning-based framework integrating variational autoencoder and neural ordinary differential equation architectures to infer developmental pseudotime and transcriptomic vector fields from single-cell gene-expression data [23]. SHG, PR-loHF, and loHF cells were extracted and analyzed using their normalized gene-expression profiles. The scTour model was trained to obtain cell-level pseudotime estimates and latent representations, and the inferred transcriptomic vector field was projected onto the low-dimensional embedding to visualize the predicted direction of cell-state progression. The relative distribution of SHG, PR-loHF, and loHF cells along the inferred scTour pseudotime and vector field was used as an additional, independent assessment of their transcriptional relationships.

Developmental potential was further assessed using CytoTRACE (version [0.3.3]) [24]. CytoTRACE scores were calculated from the number and expression patterns of genes detected in each cell, with higher scores indicating a less differentiated state and greater developmental potential. The resulting scores were compared among SHG, PR-loHF, and loHF populations and visualized on the Seurat-derived UMAP.

### 2.5. Cell-Cycle Analysis

Cell-cycle states were inferred using phase-specific marker gene sets for the G1, S, G2, and M phases, which were compiled from previously published cell-cycle markers and the Cyclebase database [25]. Module scores for each cell-cycle phase were calculated for individual cells using the AddModuleScore function implemented in Seurat. For each phase, the score was calculated as the difference between the average expression of the corresponding cell-cycle marker genes and that of randomly selected control genes with comparable expression levels. Each cell was assigned to the phase with the highest module score, whereas cells lacking detectable expression of cell-cycle-associated marker genes were classified as non-cycling. The proportions of cells in each cell-cycle state were subsequently compared among the epithelial cell subpopulations.

### 2.6. Differential Expression and Functional Enrichment Analyses

Differentially expressed genes among SHG, PR-loHF, and loHF cells were identified using the model-based analysis of single-cell transcriptomics method implemented in Seurat [26,27]. Genes with an absolute log2 fold change of ≥0.5, an adjusted *p*-value of ≤0.05, and detectable expression in at least 10% of cells in the target population were considered significantly differentially expressed.

Gene Ontology, Kyoto Encyclopedia of Genes and Genomes, and Reactome pathway enrichment analyses were performed using a hypergeometric test [28,29,30]. Genes detected in the analyzed cell populations were used as the background gene set. GO terms with a Bonferroni-corrected *p*-value of ≤0.05 were considered significantly enriched, whereas KEGG and Reactome pathways with a false discovery rate-adjusted *p*-value or q-value of ≤0.05 were considered significant.

### 2.7. Transcription Factor Regulatory Network Analysis

Transcription factor regulatory networks were inferred using SCENIC (version [1.3.1]) [31]. First, GENIE3 was used to identify co-expression relationships between transcription factors and their potential target genes [32]. Transcription factor–target gene pairs with an importance measure greater than 0.001 were retained, and co-expression modules containing at least 20 genes were included in subsequent analyses.

Regulons were then identified using RcisTarget based on motif enrichment within the cisTarget databases covering regions 500 bp upstream of the transcription start site and ±10 kb around the transcription start site. Motifs with a normalized enrichment score greater than 3 were retained, and genes supported by the corresponding transcription factor-binding motifs were defined as regulon targets. Regulon activity in individual cells was quantified using AUCell by calculating the area under the recovery curve for each regulon. Regulon activity scores were subsequently summarized and compared among SHG, PR-loHF, and loHF cells and visualized using heatmaps. Transcription factor–target gene regulatory networks were visualized using Cytoscape (version [3.10]) [33].

### 2.8. Cell–Cell Communication Analysis

Cell–cell communication was inferred using CellChat (version 2.1.0) based on the normalized single-cell gene-expression matrix and epithelial cell annotations [34]. Ligand–receptor interactions were identified using the CellChatDB database, and communication probabilities between cell populations were inferred according to the expression of ligands in sender cells and their corresponding receptors in receiver cells. Statistical significance of ligand–receptor interactions was assessed using the permutation-based framework implemented in CellChat, and interactions with a *p* value ≤ 0.05 were considered significant.

Significant ligand–receptor interactions were subsequently used to characterize predicted communication among SHG-associated, PR-loHF, loHF-associated, and other epithelial cell populations. The number and strength of predicted interactions were evaluated to assess overall communication patterns, and representative ligand–receptor pairs associated with PR-loHF were visualized.

To evaluate signaling at the pathway level, communication probabilities of ligand–receptor interactions belonging to the same signaling pathway were aggregated to estimate pathway-specific communication probabilities. Predicted outgoing and incoming signaling patterns of PR-loHF were compared across signaling pathways, and pathway-specific communication networks were visualized for selected pathways, including COLLAGEN, LAMININ, PTN, NOTCH, and WNT signaling. These analyses were interpreted as predictions of potential intercellular communication based on ligand–receptor expression rather than direct measurements of signaling activity.

## 3. Results

### 3.1. Histological Remodeling of Hair Follicles During the Hair Cycle

**Figure 1:**
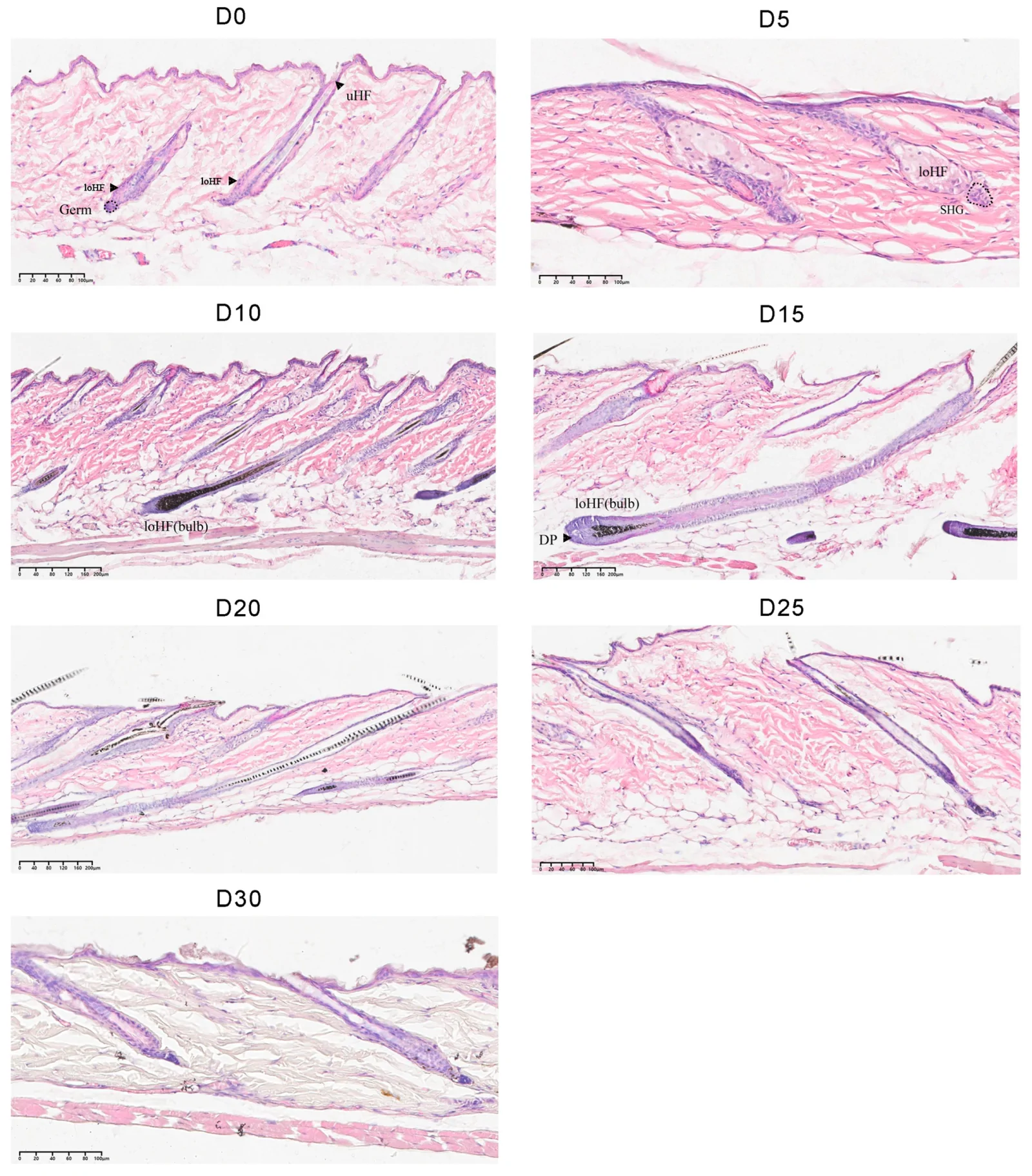
Histological changes in mouse hair follicles throughout the hair cycle.

H&E staining was performed on mouse dorsal skin collected at days 0, 5, 10, 15, 20, 25, and 30 to characterize the morphological changes occurring throughout the hair cycle (Figure 1). At day 0, the hair follicles were relatively short and located within the upper dermis, displaying morphological features consistent with a resting or early activation state. By day 5, the follicular epithelium had begun to expand and extend downward, and a morphologically distinguishable secondary hair germ (SHG) compartment could be observed adjacent to the dermal papilla.

Between days 5 and 10, the hair follicles underwent marked elongation and progressively extended into the deeper dermis. During this period, the morphologically distinguishable SHG compartment gradually became less apparent, as the lower portion of the follicle continued to expand and remodel. By day 10, a discrete SHG compartment could no longer be reliably distinguished in routine H&E-stained sections, while the developing lower hair follicle continued to elongate.

At days 15 and 20, the follicles displayed the elongated morphology characteristic of active anagen, with well-developed lower follicular structures extending into the subcutaneous tissue. Subsequently, follicular regression became apparent at day 25, followed by shortening of the follicles and re-establishment of a resting-like morphology by day 30.

The progressive loss of morphological visibility of the SHG compartment between days 5 and 10 occurred concurrently with the expansion of the developing lower hair follicle. These temporally coordinated morphological changes suggested that SHG-associated epithelial cells may undergo transcriptional and cellular state remodeling during early lower follicle development. This observation prompted us to further investigate the transcriptional relationship between SHG-associated and lower hair follicle-associated states and to determine whether a transcriptionally defined intermediate state exists in this transition.

### 3.2. Identification of a Proliferative Lower Hair Follicle-like Cell State During Early Anagen

H&E staining showed that, between days 5 and 10 of anagen, the follicular epithelium rapidly extended into the deeper dermis, while the morphologically distinguishable secondary hair germ (SHG) compartment progressively became less distinct as the developing lower hair follicle expanded. This period may therefore represent a transitional window during early lower hair follicle development. To characterize the cellular states and transcriptional changes underlying this histological remodeling, we reanalyzed single-cell RNA sequencing data from mouse dorsal skin collected on day 7 of anagen, a time point within this transitional window.

**Figure 2:**
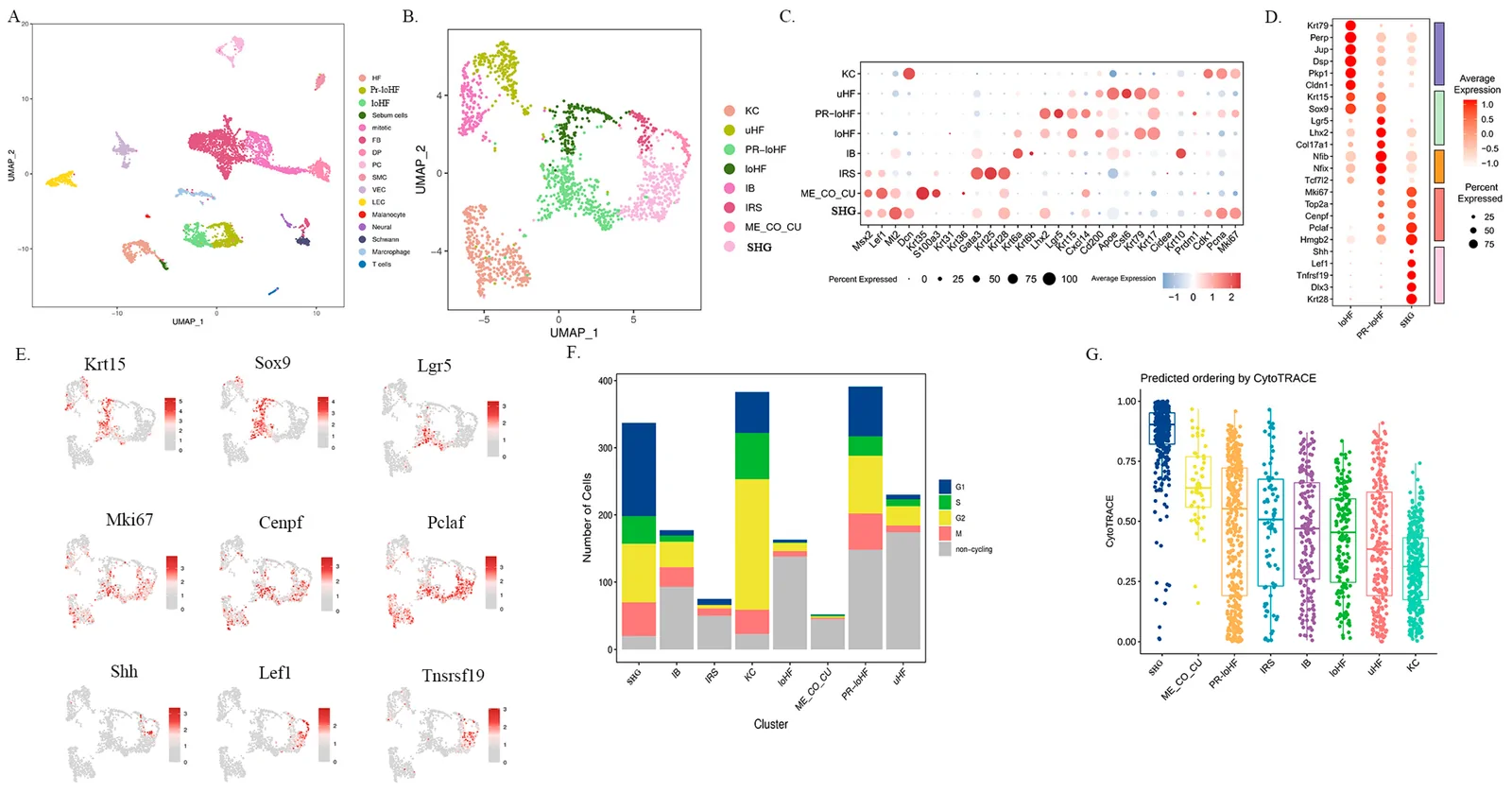
Identification and characterization of a proliferative lower hair follicle-like epithelial population in day-7 anagen skin. (**A**) UMAP visualization of all analyzed skin cells showing major cell populations. (**B**) UMAP visualization of re-clustered hair follicle-associated epithelial cells showing epithelial subclusters, including KC, uHF, loHF, PR-loHF, SHG, IB, IRS and ME_CO_CU. (**C**) Dot plot showing canonical marker gene expression used for epithelial subcluster annotation. (**D**) Dot plot showing selected marker genes across loHF, PR-loHF and SHG cells. (**E**) Feature plots showing representative lower hair follicle/progenitor markers, proliferation-related markers and SHG-associated markers. (**F**) Cell-cycle phase distribution across epithelial subclusters. (**G**) CytoTRACE analysis showing predicted developmental potential across epithelial subclusters.

Unsupervised clustering and UMAP visualization subsequently identified multiple transcriptionally distinct cell populations, including keratinocytes and hair follicle epithelial cells, fibroblasts, vascular endothelial cells, lymphatic endothelial cells, macrophages, melanocytes, dermal papilla cells, Schwann cells, neural cells, smooth muscle cells, and T cells (Figure 2A). The major skin cell populations were annotated based on canonical marker genes and cluster-specific expression patterns.

To further resolve the heterogeneity of the hair follicle epithelium, keratinocytes and hair follicle epithelial cells were extracted from the complete dataset and independently reclustered at a resolution of 0.6. Eight epithelial subpopulations were identified, including keratinocytes (KC), upper hair follicle cells (uHF), lower hair follicle cells (loHF), proliferative lower hair follicle-like cells (PR-loHF), secondary hair germ-associated cells (SHG), inner bulge cells (IB), inner root sheath cells (IRS), and medulla/cortex/cuticle cells (ME_CO_CU) (Figure 2B). These epithelial subpopulations exhibited distinct marker-gene expression profiles supporting their respective transcriptomic annotations (Figure 2C).

We next focused on the transcriptional relationship among SHG, PR-loHF, and loHF cells. PR-loHF expressed hair follicle stem/progenitor-associated genes, including *Krt15*, *Sox9*, *Lgr5*, *Lhx2*, and *Col17a1*, while simultaneously exhibiting prominent expression of proliferation-associated genes, such as *Mki67*, *Top2a*, *Cenpf*, *Pclaf*, and *Hmgb2*. In contrast, SHG-associated cells showed relatively higher expression of anagen activation- and developmental-associated genes, including *Shh*, *Lef1*, *Tnfrsf19*, and *Dlx3* (Figure 2D). Thus, PR-loHF displayed a combined transcriptional profile characterized by hair follicle progenitor/lower follicle-associated features together with an active proliferative program.

Feature plots further illustrated the expression patterns of representative genes across the epithelial transcriptional landscape (Figure 2E). Hair follicle progenitor- and lower follicle-associated markers were preferentially detected in the loHF and PR-loHF reions, whereas SHG-associated genes were enriched in the SHG-associated region. Proliferation-associated genes were strongly expressed in SHG-associated cells and remained prominently expressed in PR-loHF, whereas their expression was reduced in loHF-associated cells. This pattern suggests that PR-loHF retains an active proliferative program while progressively acquiring lower hair follicle-associated transcriptional features (Figure 2E).

Consistent with these transcriptional features, cell cycle analysis revealed distinct cell-cycle phase distributions among SHG-associated, PR-loHF, and loHF-associated cells, with substantial cycling activity retained in PR-loHF (Figure 2F). These results indicate that PR-loHF retains an active proliferative program while simultaneously exhibiting lower hair follicle-associated transcriptional features. CytoTRACE analysis further revealed heterogeneity in predicted developmental potential across the epithelial subpopulations, with PR-loHF exhibiting a predicted developmental state distinct from those of SHG-associated and loHF cells (Figure 2G). Because these epithelial subpopulations represent distinct follicular lineages, differences in CytoTRACE scores across all clusters were not interpreted as a single developmental hierarchy. Collectively, these results characterize PR-loHF as a transcriptionally distinguishable proliferative intermediate state that retains cell-cycle activity while exhibiting lower hair follicle-associated features during early anagen. These findings provided a basis for further investigating its position within the inferred transcriptional continuum between SHG-associated and loHF-associated states.

### 3.3. Convergent Trajectory Analyses Support an Inferred SHG-to-PR-loHF-to-loHF Transition During Early Anagen

**Figure 3:**
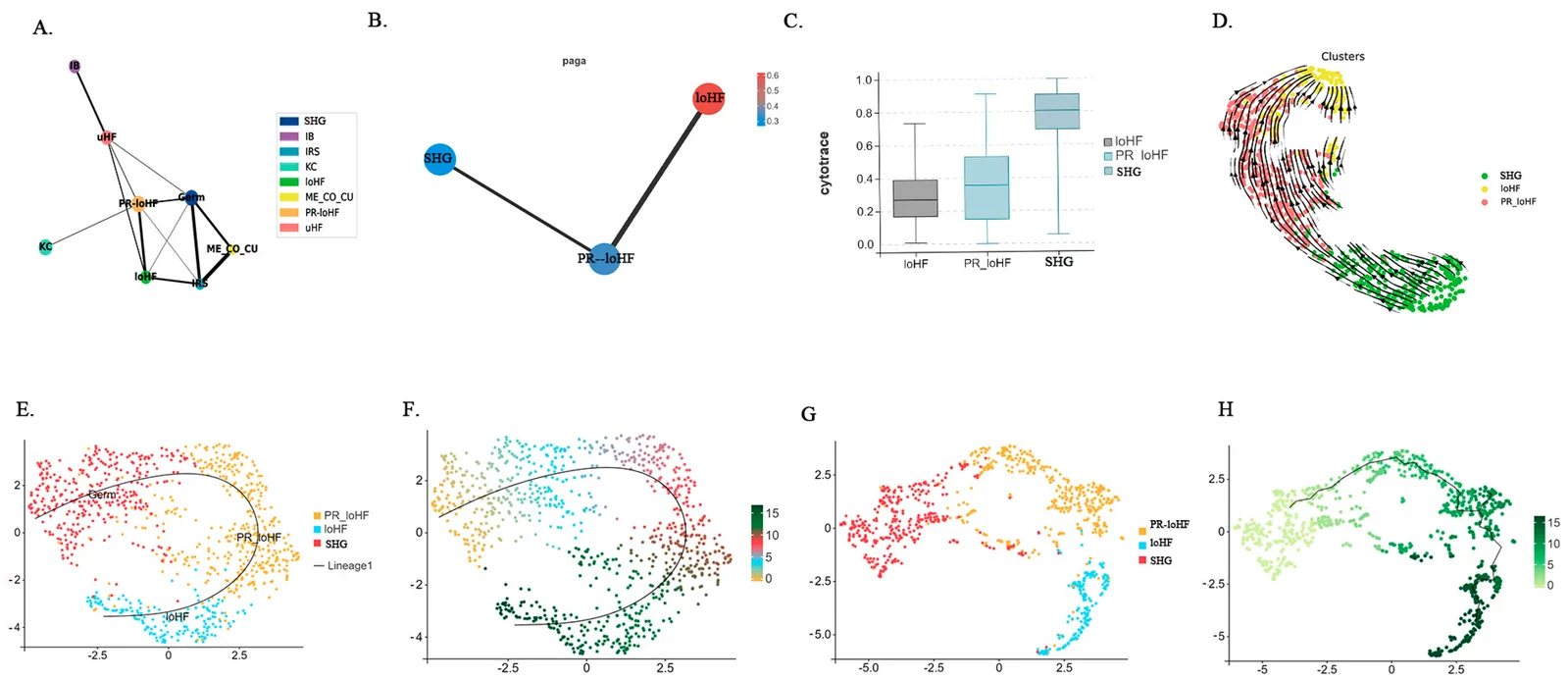
Convergent trajectory analyses support an inferred transcriptional progression from SHG-associated through PR-loHF to loHF-associated states during early anagen. (**A**) PAGA network showing transcriptional connectivity among the epithelial cell populations. Nodes represent individual epithelial populations, and edge thickness represents connectivity strength. (**B**) Focused PAGA network showing a continuous SHG–PR-loHF–loHF connection, with PR-loHF directly linking SHG-associated and loHF cells. (**C**) CytoTRACE score distributions among SHG, PR-loHF, and loHF cells. SHG-associated cells exhibited the highest developmental potential, PR-loHF cells displayed an intermediate state, and loHF cells showed the lowest developmental potential. (**D**) scTour-inferred transcriptomic vector field showing the predicted direction of cell-state dynamics among SHG-associated, PR-loHF, and loHF-associated cells. The inferred vector field was consistent with progression from the SHG-associated state through PR-loHF toward the loHF-associated state. (**E**,**F**) Slingshot trajectory reconstruction and pseudotime ordering showing a continuous trajectory across SHG-associated, PR-loHF, and loHF-associated cells, with SHG-associated cells positioned at one end, PR-loHF in an intermediate region, and loHF-associated cells at the opposite end. (**G**,**H**) Monocle 3 trajectory reconstruction and pseudotime ordering independently supported the progressive transcriptional ordering of SHG-associated, PR-loHF, and loHF-associated cells. The agreement between Slingshot and Monocle 3 supports PR-loHF as an intermediate transcriptional state within the inferred SHG-associated-to-loHF-associated continuum.

To investigate the transcriptional relationship among SHG-associated, PR-loHF, and loHF cells during early anagen, we integrated graph-based connectivity, developmental potential, and multiple trajectory inference approaches. PAGA revealed a continuous transcriptional connection linking SHG-associated, PR-loHF, and loHF cells, with PR-loHF positioned directly between the SHG-associated and loHF states (Figure 3A,B). This topology was consistent with PR-loHF occupying an intermediate position within the transcriptional continuum between SHG-associated and loHF-associated states.

CytoTRACE analysis further revealed differences in predicted developmental potential among the three transcriptional states. Because CytoTRACE scores primarily reflect transcriptomic features associated with developmental potential and are not intended to define direct lineage relationships among distinct epithelial lineages, interpretation was restricted to SHG-associated, PR-loHF, and loHF-associated states. SHG-associated cells exhibited the highest CytoTRACE scores, indicating the greater predicted developmental potential, whereas loHF-associated cells displayed comparatively lower scores. PR-loHF cells showed intermediate CytoTRACE scores between SHG-associated and loHF cells (Figure 3C). This progressive change in predicted developmental potential was consistent with PR-loHF occupying an intermediate position between SHG-associated and loHF-associated states.

Cellular dynamics were further evaluated using scTour, which infers transcriptomic vector fields and pseudotemporal relationships from single-cell gene-expression data. The scTour-inferred vector field showed a directional pattern extending from the SHG-associated region through PR-loHF toward the loHF-associated region (Figure 3D). This predicted directional pattern provided additional computational support for the intermediate position of PR-loHF within the inferred transcriptional progression.

Slingshot reconstructed a continuous trajectory extending from SHG-associated cells, passing through PR-loHF cells, and terminating in the loHF population (Figure 3E,F). SHG-associated cells were concentrated at the beginning of pseudotime, PR-loHF cells occupied the intermediate region of the lineage, and loHF cells were enriched toward later pseudotime. This ordered distribution further supported PR-loHF as an intermediate transcriptional state within the inferred progression from SHG-associated toward loHF-associated states.

Consistent with the Slingshot results, Monocle 3 independently reconstructed the same directional trajectory from SHG-associated through PR-loHF to loHF cells (Figure 3G,H). The learned principal graph extended continuously across the SHG-associated, PR-loHF, and loHF-associated regions, with the three states showing a progressive ordering along pseudotime. The concordant pseudotime organization obtained using Slingshot and Monocle 3 indicated that the inferred transcriptional ordering was not dependent on a single trajectory inference method.

Collectively, PAGA, CytoTRACE, scTour, Slingshot, and Monocle 3 consistently supported an inferred transcriptional progression from SHG-associated cells through PR-loHF toward loHF-associated cells. These results establish PR-loHF as a proliferative intermediate transcriptional state within the inferred continuum between SHG-associated and loHF-associated states during early anagen. Importantly, these computational analyses support transcriptional-state relationships and predicted directionality but do not by themselves establish direct lineage relationships among the three states.

### 3.4. PR-loHF Combines Proliferative Features with Lower Hair Follicle-Associated Remodeling Programs

Having identified PR-loHF as an intermediate state within the inferred SHG-associated–PR-loHF–loHF-associated transcriptional continuum, we next examined the transcriptional and functional programs characterizing this intermediate state. Differential expression analysis revealed marked transcriptional differences between PR-loHF and the two adjacent cell states along the trajectory.

**Figure 4:**
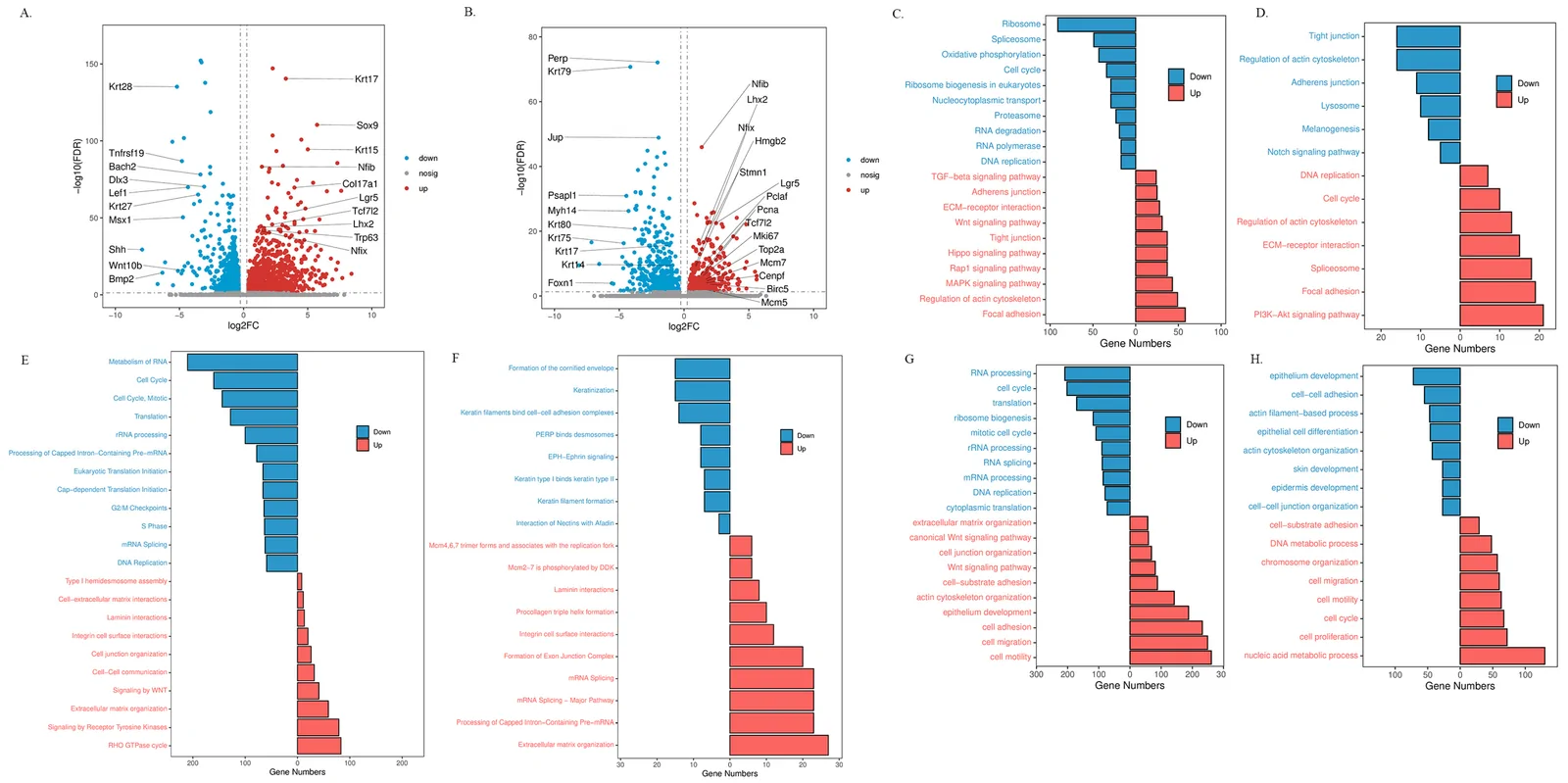
Differential expression and functional enrichment analyses define PR-loHF as a proliferative and remodeling intermediate transcriptional state between SHG-associated and loHF-associated states. (**A**) Volcano plot showing differentially expressed genes between PR-loHF and SHG-associated cells. (**B**) Volcano plot showing differentially expressed genes between PR-loHF and loHF cells. (**C**,**D**) KEGG pathway enrichment analysis of differentially expressed genes in PR-loHF versus SHG and PR-loHF versus loHF comparisons. (**E**,**F**) Reactome pathway enrichment analysis showing biological pathways associated with PR-loHF-, SHG- and loHF-enriched genes. (**G**,**H**) GO biological process enrichment analysis showing functional programs associated with epithelial proliferation, adhesion, ECM organization and differentiation-related processes.

Compared with SHG-associated cells, PR-loHF cells showed increased expression of lower hair follicle stem/progenitor-associated genes, including Krt15, Sox9, Lgr5, Lhx2, Col17a1, Tcf7l2, Nfib, and Nfix. In contrast, SHG-associated cells preferentially expressed activation and hair lineage-associated genes, including Shh, Wnt10b, Bmp2, Lef1, Dlx3, Tnfrsf19, Msx1, Krt27, and Krt28 (Figure 4A). These expression differences are consistent with PR-loHF occupying an intermediate transcriptional state characterized by reduced SHG-associated features and increased lower hair follicle-associated features.

KEGG, Reactome, and GO enrichment analyses further defined this transcriptional shift. Genes elevated in SHG-associated cells were primarily associated with ribosome biogenesis, RNA processing, translation, oxidative phosphorylation, DNA replication, and cell-cycle progression. By contrast, genes elevated in PR-loHF were enriched in Wnt, TGF-β, Hippo, MAPK, Rap1, and receptor tyrosine kinase signaling, together with extracellular matrix organization, ECM–receptor interaction, focal adhesion, adherens and tight junctions, laminin and integrin interactions, actin cytoskeleton regulation, cell migration, and epithelial development (Figure 4C,E,G). These results indicate that the transcriptional distinction between SHG-associated and PR-loHF states involves reduced predominance of biosynthetic and cell-cycle-associated programs together with increased representation of adhesion, signaling, and tissue-remodeling programs associated with lower follicle development.

PR-loHF also differed markedly from loHF cells. Relative to loHF, PR-loHF strongly expressed proliferation- and DNA replication-associated genes, including Mki67, Top2a, Pcna, Pclaf, Cenpf, Birc5, Mcm5, Mcm7, Stmn1, and Hmgb2, as well as the stem/progenitor regulators Lgr5, Lhx2, Nfib, Nfix, and Tcf7l2. In contrast, loHF cells displayed higher expression of epithelial structural and differentiation-associated genes, including Perp, Jup, Krt79, Krt80, Krt75, Krt17, Krt14, Myh14, and Foxn1 (Figure 4B).

Consistent with these gene-level differences, pathways enriched in PR-loHF relative to loHF included DNA replication, cell cycle, chromosome organization, RNA splicing and processing, PI3K–Akt signaling, focal adhesion, ECM–receptor interaction, laminin and integrin interactions, extracellular matrix organization, cell migration, and cell proliferation. Conversely, loHF-enriched genes were associated with keratinization, cornified envelope formation, keratin filament organization, desmosomal interactions, epithelial differentiation, epidermal development, and cell–cell junction organization (Figure 4D,F,H).

Collectively, these analyses support the characterization of PR-loHF as a molecularly coherent intermediate transcriptional state. PR-loHF combines prominent adhesion, extracellular matrix, signaling, and morphogenetic programs associated with lower hair follicle development while retaining proliferative and nucleic acid-processing features that are comparatively reduced in loHF-associated cells. PR-loHF combines prominent adhesion, extracellular matrix, signaling, and morphogenetic programs associated with lower hair follicle development while retaining proliferative and nucleic acid-processing features that are comparatively reduced in loHF-associated cells.

### 3.5. PR-loHF Exhibits Distinct Regulatory and Predicted Cell–Cell Communication Features

**Figure 5:**
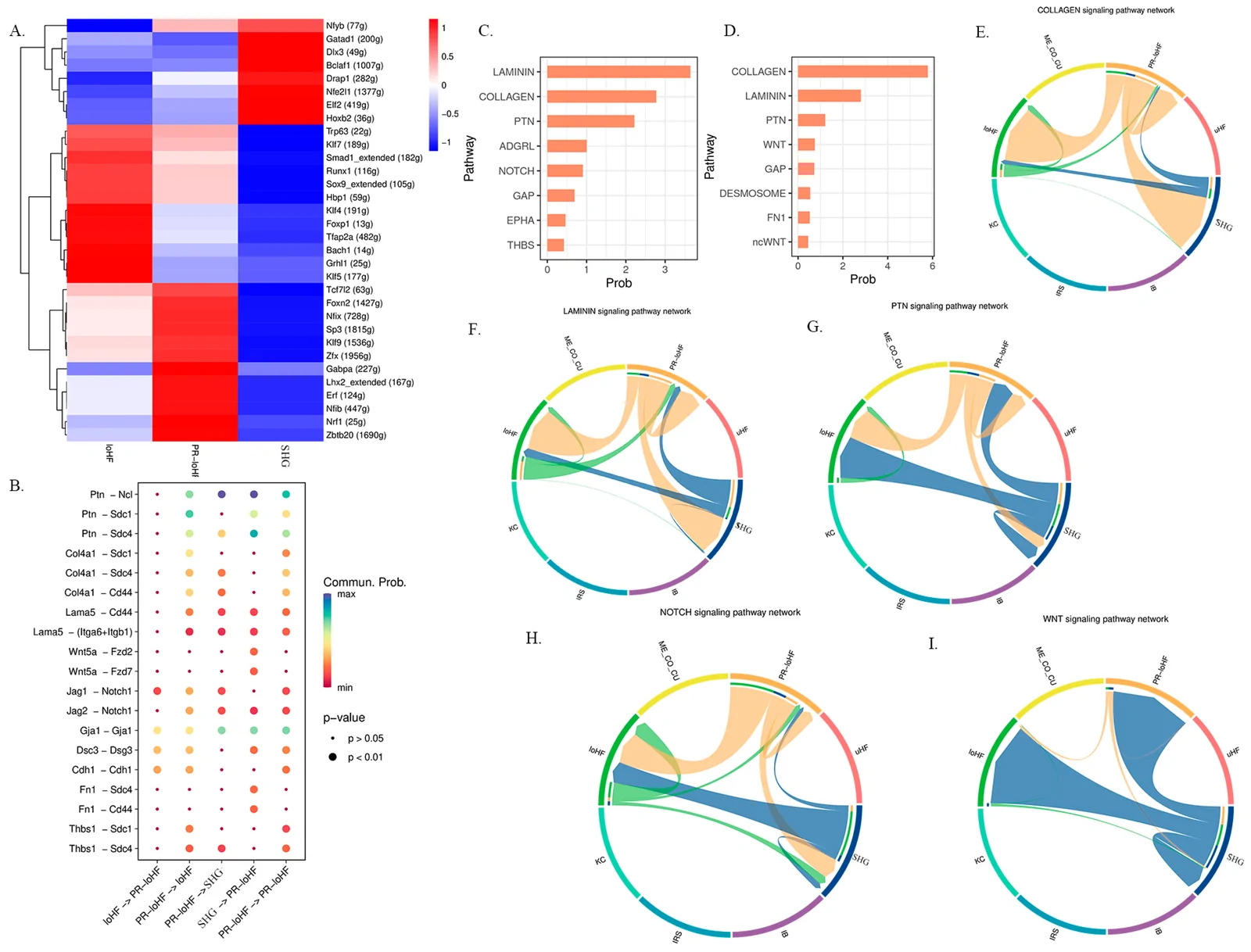
PR-loHF exhibits distinct regulatory and predicted cell–cell communication features during early anagen. (**A**) Heatmap showing selected transcription factor regulon activity across loHF, PR-loHF and SHG-associated cells. (**B**) Dot plot showing representative ligand-receptor interactions among loHF, PR-loHF and SHG-associated cells. (**C**,**D**) Pathway-level analysis showing outgoing and incoming signaling pathways associated with PR-loHF. (**E**–**I**) Circle plots showing pathway-specific communication networks for COLLAGEN, LAMININ, PTN, NOTCH and WNT signaling pathways among epithelial subclusters.

To characterize the regulatory features associated with the intermediate transcriptional state of PR-loHF, we performed SCENIC analysis and compared inferred regulon activities among SHG-associated, PR-loHF, and loHF-associated cells. The three populations exhibited clearly distinct transcription factor regulatory programs (Figure 5A). SHG-associated cells showed elevated activities of regulons including Dlx3, Nfyb, Nfe2l1, Elf2, and Hoxb2, consistent with their anagen-activated and hair lineage-associated transcriptional features. In contrast, loHF cells displayed strong activities of epithelial stem/progenitor and structural regulons, including Trp63, Runx1, Sox9, Klf4, Klf5, Foxp1, and Tfap2a.

PR-loHF cells were characterized by a distinguishable regulon activity profile involving Tcf7l2, Nfix, Nfib, Lhx2, Zfx, Sp3, Erf, and Nrf1 regulons (Figure 5A). The differential regulon activity patterns further distinguished PR-loHF transcriptionally from both SHG-associated and loHF-associated states, providing additional evidence that PR-loHF exhibits regulatory features not fully represented by either adjacent state. These regulatory features were associated with progenitor maintenance, cellular proliferation, transcriptional reorganization, and lower hair follicle-associated characteristics, consistent with the proposed intermediate transcriptional state of PR-loHF.

We next examined the signaling interactions connecting PR-loHF with SHG and loHF cells. Ligand–receptor analysis revealed extensive bidirectional communication between PR-loHF and the two adjacent cell states, together with prominent autocrine signaling within the PR-loHF population (Figure 5B). These interactions included extracellular matrix-associated ligand–receptor pairs, such as COL4A1–SDC1, COL4A1–SDC4, COL4A1–CD44, LAMA5–CD44, LAMA5–(ITGA6 + ITGB1), FN1–SDC4, FN1–CD44, and THBS1–SDC1/SDC4. Developmental and cell–cell communication signals were additionally represented by PTN–NCL/SDC1/SDC4, WNT5A–FZD2/FZD7, JAG1/2–NOTCH1, GJA1–GJA1, DSC3–DSG3, and CDH1–CDH1 interactions.

Analysis of the outgoing signaling profile identified LAMININ, COLLAGEN, and PTN as the dominant pathways produced by PR-loHF, followed by ADGRL, NOTCH, GAP, EPHA, and THBS signaling (Figure 5C). Conversely, PR-loHF predominantly received COLLAGEN, LAMININ, and PTN signals, with additional contributions from WNT, GAP, DESMOSOME, FN1, and non-canonical WNT signaling (Figure 5D). These results demonstrate that PR-loHF simultaneously functions as a signal-producing and signal-receiving population within the regenerating follicular epithelium.

Pathway-specific communication networks further revealed extensive interactions among PR-loHF, SHG-associated, loHF, and other epithelial populations through COLLAGEN, LAMININ, PTN, NOTCH, and WNT signaling (Figure 5E–I). PR-loHF occupied a central position in these networks and maintained strong signaling connections with both SHG-associated and loHF cells. In particular, COLLAGEN and LAMININ signaling established an extracellular matrix and adhesion environment linking the three populations, whereas PTN, NOTCH, and WNT signaling provided developmental cues associated with cell-state progression.

Collectively, these findings further support PR-loHF as a transcriptionally distinguishable intermediate state characterized by a specific regulon activity profile and predicted cell–cell communication features within the SHG-associated–PR-loHF–loHF-associated transcriptional continuum. Its regulon activity profile, together with predicted extracellular matrix-, adhesion-, and development-associated signaling interactions, provides additional molecular characterization of the PR-loHF state and is consistent with transcriptional remodeling occurring between SHG-associated and lower hair follicle-associated states during early anagen.

## 4. Discussion

This study began with a clear morphological observation. H&E staining showed that the secondary hair germ (SHG) compartment remained distinguishable during early anagen. The morphologically recognizable SHG compartment became progressively less apparent between days 5 and 10. After day 10, it could no longer be reliably distinguished as a discrete structure in routine H&E sections. At the same time, the lower portion of the hair follicle rapidly expanded and extended downward. These morphological changes occurred concurrently. Thus, the reduced morphological visibility of the SHG compartment should not be interpreted as disappearance of SHG-associated cells. Rather, these concurrent morphological changes are consistent with progressive epithelial and transcriptional remodeling during lower hair follicle development.

To investigate the transcriptional states associated with this transitional interval, we analyzed single-cell RNA-sequencing data from mouse skin collected on anagen day 7. Day 7 lies within the critical interval between days 5 and 10. During this period, the morphologically recognizable SHG compartment becomes less distinct, whereas the developing lower follicle rapidly expands. This time point was therefore suitable for examining transcriptional heterogeneity associated with this morphological transition. Previous studies have shown that the secondary hair germ is activated early during the telogen-to-anagen transition. Activated SHG cells generate progeny and contribute to anagen hair follicle growth [5,7,35]. In addition, proliferative Lgr5-positive cells in the secondary hair germ and lower outer root sheath have long-term regenerative potential [8]. However, the transcriptional intermediate states accompanying the progression from SHG-associated toward lower hair follicle-associated states have not been fully resolved. Previous single-cell studies revealed extensive heterogeneity among hair follicle epithelial cells, but the transcriptional features of intermediate states associated with this process remain incompletely characterized [12,13].

After reclustering the hair follicle epithelial cells, we identified a proliferative transcriptional state between SHG-associated and loHF states. We termed this state PR-loHF. PR-loHF highly expressed cell-cycle-associated genes, including *Mki67*, *Top2a*, *Cenpf*, *Pclaf*, and *Hmgb2*. It also expressed hair follicle stem/progenitor and lower follicle-associated genes, including *Krt15*, *Sox9*, *Lgr5*, *Lhx2*, and *Col17a1* [8,36,37,38]. Cell cycle analysis further indicated strong cycling activity in SHG-associated cells, with substantial proliferative activity retained in PR-loHF and comparatively reduced activity in loHF-associated cells. These findings indicate that PR-loHF exhibits a combination of transcriptional features not fully represented by either adjacent state. Together, these features support the characterization of PR-loHF as a transcriptionally distinguishable proliferative intermediate state.

Trajectory analyses further evaluated the transcriptional position of PR-loHF. PAGA showed strong transcriptional connectivity between PR-loHF and both SHG and loHF cells. PR-loHF occupied an intermediate position within this continuous transcriptional network. CytoTRACE further placed PR-loHF at an intermediate level of predicted developmental potential between SHG-associated and loHF-associated states. In addition, the scTour-inferred transcriptomic vector field showed a directional pattern extending from the SHG-associated region through PR-loHF toward the loHF-associated region. Monocle 3 and Slingshot independently inferred concordant pseudotime patterns. Both analyses positioned SHG-associated cells preferentially toward one end of pseudotime, PR-loHF in an intermediate region, and loHF-associated cells toward the opposite end. The agreement among these methodologically distinct computational analyses strengthens the robustness of the inferred transcriptional ordering. Within the single-cell trajectory framework, our results therefore support an inferred SHG-associated → PR-loHF → loHF progression. Within this framework, PR-loHF occupies a proliferative intermediate transcriptional state.

This finding extends the transcriptional characterization of SHG-associated epithelial remodeling during early anagen [5,7,35]. Earlier studies showed that activated SHG cells produce progeny that contributes to regenerating hair follicle structures. However, the specific states of these progenies were not fully resolved at single-cell resolution. Our results suggest that the transition toward a loHF-associated transcriptional state may involve an intermediate PR-loHF-like state rather than an abrupt transcriptional change. PR-loHF retains proliferative features while exhibiting transcriptional programs associated with epithelial remodeling and lower follicle development. In contrast, loHF-associated cells exhibit stronger structural and differentiation-associated programs. PR-loHF therefore represents an intermediate transcriptional state associated with the transition between SHG activation and lower follicle formation.

Differential expression and functional enrichment analyses further supported this transcriptional-state model. SHG-associated cells showed prominent activation, biosynthetic, DNA-replication, and cell-cycle-related programs. PR-loHF PR-loHF retained proliferative features while showing stronger cell-adhesion, extracellular-matrix, signaling, and cytoskeletal-remodeling programs. In contrast, loHF cells showed stronger epithelial structural, junctional, and differentiation-associated programs. These differences are consistent with a continuous transcriptional transition. Within the inferred continuum, SHG-associated cells are characterized by strong activation and proliferative programs, PR-loHF combines retained proliferative activity with tissue-remodeling features, and loHF-associated cells exhibit stronger structural and differentiation-related programs.

Notably, PR-loHF was enriched not only in proliferation-related pathways but also in extracellular matrix and basement membrane-associated programs. Lower follicle formation requires more than an increase in cell number. It also requires cell adhesion, migration, rearrangement, and interaction with the basement membrane. PR-loHF was enriched in ECM–receptor interaction, focal adhesion, laminin–integrin interaction, and actin cytoskeleton regulation. Laminin, integrin, and epithelial–mesenchymal interactions are important for hair follicle structure, basement membrane attachment, and follicular morphogenesis [39,40,41]. These findings suggest that PR-loHF transcriptional state is associated with both proliferative activity and structural-remodeling programs relevant to lower follicle development.

SCENIC and cell–cell communication analyses provided additional support for this interpretation. PR-loHF showed a regulatory profile that differed from those of Germ and loHF cells. Regulons associated with *Tcf7l2*, *Nfib*, *Nfix*, and *Lhx2* displayed characteristic activity in PR-loHF. LHX2-, TCF/LEF-, and NFIB-associated programs have been linked to hair follicle stem-cell maintenance, epithelial development, and regulation of the follicular niche [36,42,43]. *Nfix* may represent an additional candidate regulator of the PR-loHF state. Cell–cell communication analysis also showed strong COLLAGEN and LAMININ signaling in PR-loHF. WNT, NOTCH, PTN, FN1, and THBS signaling pathways were also detected. WNT and NOTCH are important regulators of follicular progenitor activity and epithelial differentiation [44,45]. COLLAGEN and LAMININ signaling are closely associated with cell adhesion and basement membrane organization [39,40,41]. The prominent PTN signaling identified here may represent an additional signaling feature associated with the PR-loHF state. Together, these findings indicate that PR-loHF is an actively regulated cell state rather than a passive point along the trajectory.

Our results also provide a transcriptional framework for the morphological changes observed in the SHG compartment. The inability to distinguish a discrete SHG compartment by routine HE staining at later stages does not necessarily indicate elimination of cells associated with the earlier SHG compartment. Rather, these morphological changes are compatible with progressive epithelial remodeling and changes in transcriptional state during lower follicle development. Thus, the reduced morphological distinction of the SHG compartment and the expansion of the developing lower follicle may represent concurrent aspects of the same regenerative process. Our single-cell analyses suggest that this regenerative interval is accompanied by a transcriptional continuum in which PR-loHF occupies an intermediate position between SHG-associated and loHF-associated states.

This study has several limitations. First, the single-cell analysis was mainly based on anagen day-7 data and therefore provides only a transcriptional snapshot of a dynamic regenerative process. Single-cell datasets collected at multiple closely spaced stages of early anagen would be required to define the temporal emergence, persistence, and disappearance of the PR-loHF state more directly. Second, routine H&E staining provides morphological context but cannot determine the spatial localization of the transcriptionally defined PR-loHF state or directly trace SHG-derived cells. Third, pseudotime and scTour analyses provide computational estimates of transcriptional-state relationships and directionality but cannot establish direct lineage relationships. Future studies using SHG-specific in vivo lineage tracing will be required to determine whether cells exhibiting the PR-loHF transcriptional state represent a defined intermediate along this lineage in vivo. Spatial transcriptomics, RNA in situ hybridization, and marker-based immunofluorescence will also be needed to determine the precise anatomical localization of PR-loHF and its spatial relationship with SHG and lower follicular compartments. Finally, PR-loHF was identified in a single published scRNA-seq dataset, and independent replication will be important for determining the reproducibility and generalizability of this transcriptional state.

## 5. Conclusions

In conclusion, our morphological observations identified days 5–10 as a critical transitional window during which the secondary hair germ (SHG) compartment became less morphologically distinct while the developing lower follicle expanded. Analysis of day-7 single-cell data supported an inferred SHG-associated → PR-loHF → loHF-associated transcriptional progression. Within this inferred transcriptional continuum, PR-loHF represents a proliferative intermediate state characterized by both active cell-cycle programs and lower hair follicle-associated transcriptional features. PR-loHF combines retained proliferative activity with transcriptional reorganization and extracellular matrix- and adhesion-associated remodeling programs. The convergence of morphological, transcriptomic, trajectory, functional, and regulatory analysis supports PR-loHF as a transcriptionally defined intermediate state associated with the transition from SHG-associated toward lower hair follicle-associated states during early anagen.

## Data Availability

The single-cell RNA-seq data analyzed in this study were derived from the previously published study by Wang et al. The processed gene expression matrix and corresponding metadata were obtained from the original research group upon reasonable request.
